# Supplementary material for: Fabrication and Characterization of Fast-Dissolving Films Containing Escitalopram/Quetiapine for the Treatment of Major Depressive Disorder
Source: Pharmaceutics. 2021 Jun 16;13(6):891. doi: 10.3390/pharmaceutics13060891 (PMC8234593; doi:10.3390/pharmaceutics13060891)
Supplement: Supplementary file 1 [file pharmaceutics-13-00891-s001.zip › pharmaceutics-1233368-supplementary.pdf]

pharmaceutics

MDPI

Fabrication and Characterization of Fast-Dissolving Films Containing Escitalopram/Quetiapine for the Treatment of Major Depressive Disorder

Manal E. Alkahtani, Alhassan H. Aodah, Omar A. Abu Asab, Abdul W. Basit, Mine Orlu and Essam A. Tawfik

1. Drug Loading (DL) and Entrapment Efficiency (EE%) Determination

Calibration curves of both ESC and QUE were plotted using the peak area (AUC) data generated by the developed HPLC method for known standards concentrations (Figure S1 and S2). The AUC data of the examined samples alongside the regression equations from the calibration curves were used to determine the unknown concentrations of samples. The actual drug amount (μg) was determined by dividing the concentration (μg/mL) by the volume (mL) of the SSF used. The theoretical amount of fibers was calculated using the amount of the solid materials (polymer and drug) in the total volume of spinning solution that was injected. Finally, Equations 1 and 2, in the main text, were used to calculate *DL* and *EE%*, respectively.

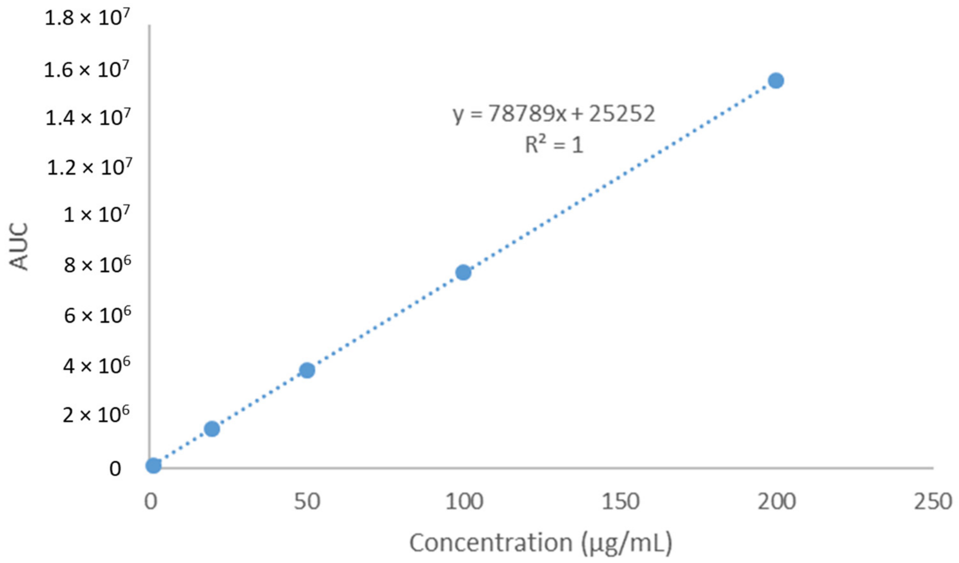

Figure S1. Calibration curve of ESC showing the regression equation and correlation coefficient (R^2).

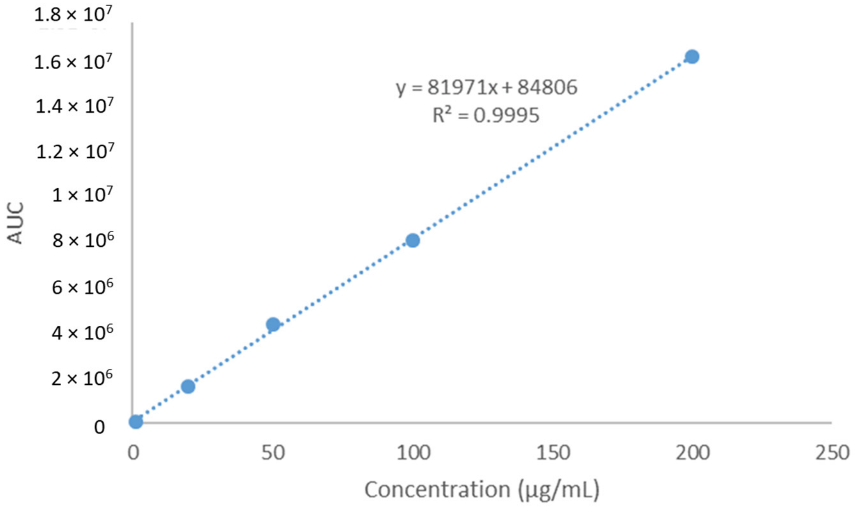

Figure S2. Calibration curve of QUE showing the regression equation and correlation coefficient (R^2).

2. High Performance Liquid Chromatography (HPLC) Method for Determining ESC and QUE in Fibers

Figure S3 shows representative chromatograms for injecting ESC and QUE in combination dissolved in either (a) SSF pH 6.8 or (b) PBS pH 7.4. The peaks in Figure S3 (a) showed good separation and a high degree of symmetry; whereas, in Figure S3 (b) the peaks showed a slightly broader shape that does not influence the separation, but displayed a degree of asymmetry with QUE peak having a tailing appearance.

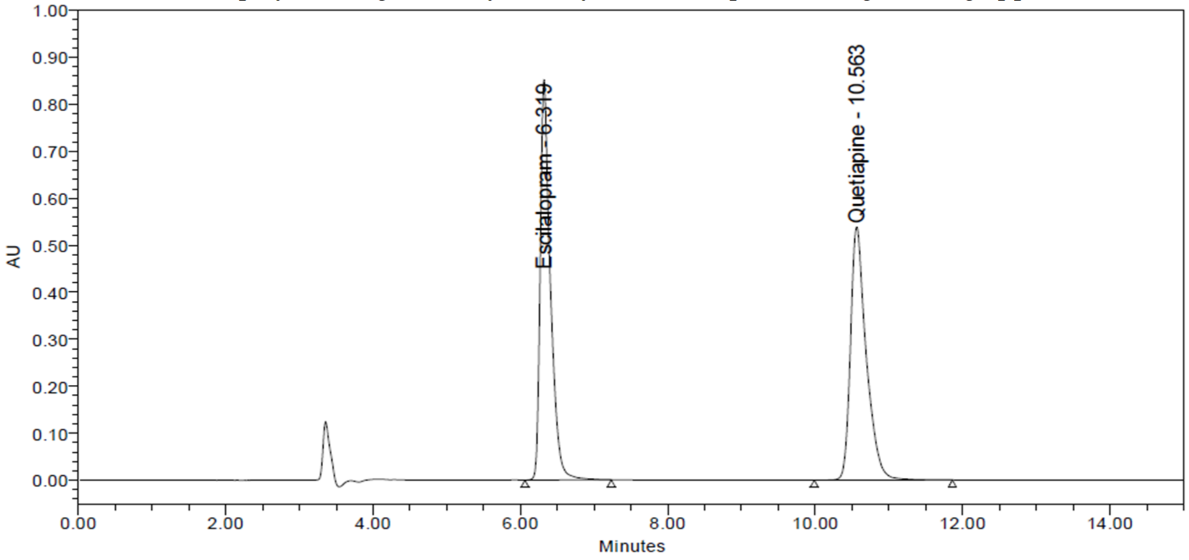

(a)

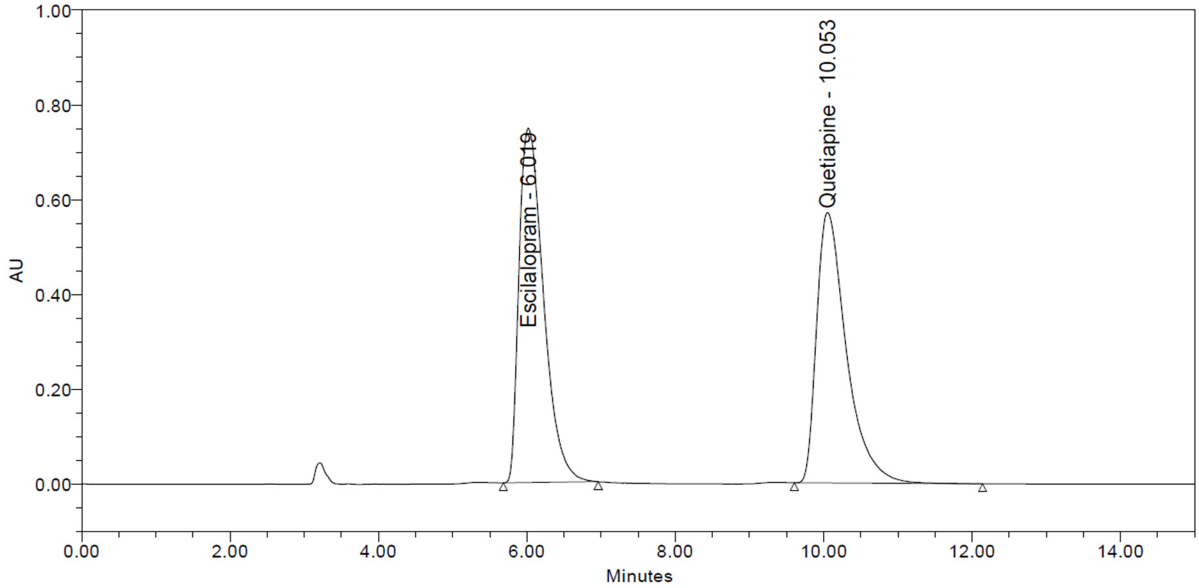

(b)

Figure S3. Representative HPLC chromatograms using (**a**) SSF extraction method of ESC (100 µg/mL) and QUE (100 µg/mL) in combination, (**b**) PBS extraction method of ESC (100 µg/mL) and QUE (100 µg/mL) in combination.
